# Establishment of a transgenic mouse to model ETV7 expressing human tumors

**DOI:** 10.1007/s11248-018-0104-z

**Published:** 2018-11-27

**Authors:** Masashi Numata, R. I. Klein Geltink, Gerard C. Grosveld

**Affiliations:** 1 Present Address: Daiichi Sankyo Co., Ltd 1-2-58 Hiromachi, Shinagawa-Ku, Tokyo, 140-8710 Japan; 2 Present Address: Department of Immunometabolism, Max Planck Institute of Immunobiology and Epigenetics, 79108 Freiburg, Germany; 3 Department of Genetics, St Jude Children’s Research Hospital, 262 Danny Thomas Place, Memphis, TN 38105 USA

**Keywords:** ETV7, ETS transcription factor, Transgenic mouse, Tumor mouse model, Leukemia

## Abstract

The ETS transcription factor ETV7 has been characterized as a hematopoietic oncoprotein, which requires cooperating mutations for its leukemogenic activity. Although the *ETV7* gene is highly conserved among vertebrates, part of the rodents, including *Mus musculus*, deleted the *Etv7* gene locus. Many human hematopoietic malignancies upregulate ETV7 expression but contrary to ETV7’s role in oncogenesis, its physiological role in normal tissues is unknown. To determine the physiological function of ETV7 in vivo and determine its role in tumorigenesis in a mouse model, we have generated an ETV7 transgenic mouse that carries a single copy of human BAC DNA containing the *ETV7* gene locus and its regulatory sequences. *ETV7* heterozygous (*ETV7Tg*^+*/WT*^) mice were fertile, normal in size and born at a normal Mendelian frequency. They had a normal blood count, did not display any gross physical or behavioral abnormalities, and were not tumor-prone. The *ETV7* expression pattern in hematopoietic cells of *ETV7Tg*^+*/WT*^ mice is very similar to that in human hematopoietic cells. To examine the oncogenic potential of ETV7 in vivo, we crossed *ETV7Tg*^+*/WT*^ mice with tumor-prone mouse models. ETV7 greatly accelerated loss of Pten (phosphatase and tensin homolog)-evoked leukemogenesis in *Pten*^*Δ/Δ*^*ETV7Tg*^+*/WT*^ mice after deletion of the conditional *Pten allele*. Consistent with this observation, ETV7 expression enhanced the colony-forming and self-renewal activities of primary myeloid *Pten*^−*/*−^ cells. In this study we established a transgenic mouse in which we can more accurately model ETV7-associated human tumorigenesis in vivo.

**Electronic supplementary material:**

The online version of this article (10.1007/s11248-018-0104-z) contains supplementary material, which is available to authorized users.

## Introduction

E26-transformation specific (ETS) transcription factors are involved in diverse biological processes including cellular proliferation, survival, differentiation, development, and transformation. We and others independently identified the ETS transcription factor ETV7, which is highly homologous to ETV6/TEL, a frequent target of chromosomal translocation in human leukemia (Fenrick et al. 2000; Poirel et al. 2000; Potter et al. 2000). Given that deletion or inactivation of ETV6 has been frequently observed in hematopoietic malignancies, ETV6 is also considered to be a tumor suppressor. In contrast, ETV7 is frequently upregulated in a variety of human cancers, including hematopoietic malignancies, in which ETV7 is overexpressed in 70% of myeloid and lymphoid leukemia. Previously we have shown that ectopic retroviral expression of ETV7 causes hematopoietic malignancies in the mouse (Cardone et al. 2005; Carella et al. 2006). More recently, we have demonstrated that morpholino knockdown of *Etv7* in zebrafish leads to loss of hemoglobin-containing red blood cells by repression of the *lanosterol synthase* (*lss*) gene, indicating that in fish ETV7 is indispensable for normal red blood cell development (Quintana et al. 2014). However, the physiological and oncogenic roles of ETV7 in mammals in vivo remain to be investigated by using an appropriate mouse model.

Despite its high level of conservation among vertebrates, the *Etv7* gene locus has been deleted in part of the rodents, including *Mus musculus*. To reverse this situation in the mouse, we have generated an ETV7 BAC transgenic mouse that carries a partial single copy of a human *ETV7* BAC DNA. Like wild-type (WT) controls ETV7 heterozygous (*ETV7Tg*^+*/WT*^ or *ETV7Tg*) mice develop normally, are not tumor-prone and have a normal lifespan. Importantly, the *ETV7* expression pattern in hematopoietic cells of *ETV7Tg*^+*/WT*^ mice was evaluated by qRT-PCR and was very similar to that in human hematopoietic cells, suggesting that our *ETV7Tg*^+*/WT*^ mouse properly reflects the tissue-specific expression of human ETV7. Based on flow cytometric analysis with antibodies specific for lymphoid, myeloid, and erythroid cell types, the cellularity and distribution of hematopoietic cells in *ETV7Tg* BM, spleen, and thymus are similar to those in WT mice. Nonetheless, *ETV7Tg* BM cells proliferated faster in long-term culture, in which ETV7 enhanced proliferation of myeloid cells compared with that of control WT myeloid cells. To examine the oncogenic potential of ETV7 in vivo, we crossed *ETV7Tg* mice with an established leukemic mouse model. We found that ETV7 greatly accelerated *Pten*^Δ/Δ^ leukemogenesis in *Pten*^*fl/fl*^*;Mx1*-*Cre;ETV7Tg*^+*/WT*^ mice. Thus, we created a valuable experimental animal model to investigate the mechanism of ETV7-associated human tumorigenesis in vivo. Moreover, our *ETV7Tg* mouse model, which faithfully recapitulates human tumors, might greatly facilitate the identification of therapeutic targets for ETV7-associated human cancer.

## Materials and methods

### Generation of ETV7 BAC transgenic mice

Linearized RP11-918H23 BAC DNA (BACPAC Resources Center), containing the human *Etv7* gene locus, was microinjected into the pronucleus of fertilized FVB mouse oocytes. Injected zygotes were transplanted into pseudo pregnant CD1 fosters. Tail biopsies of live born offspring were used to isolate genomic DNA for genotyping, using primers specific for exon 1 and 8 of human ETV7. Samples positive for both PCRs were subjected to PCR screening of the upstream and downstream sequences of ETV7 as well as the first and last exons of all open reading frames (ORFs) present within the RP11-918H23 BAC. When ETV7 was detected in tail biopsies, a fresh biopsy was obtained and subjected to fluorescent in situ hybridization (FISH) using a FITC labeled RP11-918H23 probe, to determine copy number and potential mosaicism of the founder mice. The FISH analysis was carried out by the Cytogenetic Core of St. Jude Children’s Research Hospital performed.

### RNA isolation

Cells (5 × 10^6^) were taken up in TRIzol Reagent (Invitrogen) and incubated at room temperature for 10 min. Chloroform (Fisher-Scientific) was added to facilitate phase separation during centrifugation. 1 μg glycogen (Invitrogen) was added to the aqueous phase and the DNA was precipitated using 2-propanol (Fisher Scientific). RNA pellets were washed with 75% ethanol and dissolved in nuclease-free water (Ambion). The RNA was quantitated using a Nanodrop spectrophotometer (Thermo Scientific).

### Quantitative reverse transcriptase PCR

Total RNA (5 μg) was pretreated with DNase (Invitrogen), followed by first strand cDNA synthesis, using Oligo-dT priming and the SuperScript III First Strand Synthesis System (Invitrogen). After first strand synthesis, samples were treated with RNase. Quantitative Real Time PCR amplification was performed with 1 μL cDNA, using TaqMan Gene Expression Master Mix (Applied Biosystems). The library of tissue-specific human cDNAs was purchased from Clontech. The TaqMan probe/primers set for human *Etv7* was as described previously (Kawagoe et al. 2004). 20 μL reactions were loaded in a MicroAmp Optical 96-well reaction plate (Applied Biosystems) and amplification was performed and detected using the ABI Prism 7900HT Sequence Detection System (Applied Biosystems). Samples were amplified in parallel using human or murine *HPRT* as internal control. The sets of TaqMan probes and primers for human *HPRT* were as suggested by Applied Biosystems (4326321E). The murine *HPRT* TaqMan probe and primers are as follows: probe (5′-CGAGCAAGTCTTTCAGTCCTGTCCA-3′), forward (5′-ATTATGCCGAGGATTTGGAA-3′), and reverse (5′-CCCATCTCCTTCATGACATCT-3′). Standard curves were generated using 5 μL of serially diluted standards with a starting concentration of 2.40 × 10^9^ copies. Human CD19^+^ (B-cells), CD3^+^ (T-cells), CD11b^+^CD15^+^ (Granulocytes), and CD11b^+^CD15^+^ (Monocytes) were sorted from human cord blood cells (St. Louis Cord Blood Bank) using a FACS Vantage-SE DiVa cell sorter (BD Biosciences), and the individual total RNA was purified as described above.

### Tissue staining with ETV7 antibody

Murine normal tissues were obtained from humanely euthanized animals and fixed in 10% neutral buffered formalin. Tissues were paraffinized, embedded, and 5 μm thick sections were cut. For anti-ETV7 antibody staining, the sections were dried overnight, and baked at 65 °C for 30 min. The sections on slides were incubated in citrate buffer at pH 6.0 (Invitrogen) for 15 min at 100 °C. After antigen retrieval, endogenous peroxidase activity was blocked by incubating the slides in 3% peroxide (Sigma) in methanol for 5 min. All following steps were intermitted by washing in TBS with 0.5% Tween-20. Endogenous biotin was blocked using an avidin/biotin-blocking kit (Vector Labs) followed by a 30-min protein-blocking step with Serum-Free protein block (Invitrogen) at 37 °C. Sections were incubated with anti-ETV7 antibody overnight at 4 °C. For peptide competition, undiluted antibody was incubated with ETV7 peptide at room temperature (RT) for 30 min prior to its application to the slides. Biotinylated secondary antibody (Vector Labs) was used at 6 μg/mL for 30 min at RT, followed by streptavidin-HRP (DAKO) and DAB chromogen (DAKO), following the manufacturer’s protocols. Images were acquired using 200x or 400 × magnification on a Nikon E800 microscope in the Cell Imaging Core Facility of St. Jude Children’s Research Hospital. The ETV7 polyclonal antibody (Cardone et al. 2005) was raised against the ETV7-C-terminal peptide (DRIEFKDKRPEISP) and affinity purified using the same peptide coupled to an agarose column. The antibody was recovered using glycine elution.

### FCM analysis

Mononuclear cells were freshly harvested from bone marrow (BM), thymus, and spleen of 8–12 week-old mice and immediately stained with the antibodies of interest; B220-eFluor780, CD43-PE, IgM-PE-Cy7, IgD-APC, Mac1-Alexa700, and Gr1-APC-Cy7 for bone marrow cells, CD4-PerCP-Cy5.5, CD8-Alexa700, CD25-APC, CD44-PE-Cy7, CD3-PE, cKit-APC-eFluor780, and Lin^+^ cocktail (B220, Mac1, and Gr1)-FITC for thymocytes, and B220-eFluor605, CD3-APC, Mac1-Alexa700, CD4-PE, CD8-PE-Cy7, and Gr1-APC-Cy7 for splenocytes as shown in Fig. 4. Single-cell suspensions were incubated on ice for 30 min in staining medium (SM; PBS with 5% FBS), containing 100 mg/mL human gamma globulin solution to block non-specific staining. After washing, the cells were incubated on ice for 15 min in SM containing fluorochrome-conjugated antibodies. For detection of cells undergoing apoptosis, samples were incubated at room temperature for 15 min in Annexin V binding buffer (10 mM HEPES, 0.9% NaCl, 2.5 mM CaCl_2_, and 0.1% BSA) containing Annexin V-FITC antibody. DAPI was used as a dead cell marker. All FCM analyses were carried out using a BD LSR II flow cytometer (BD Biosciences).

### *Pten*^*fl/fl*^, *Pten*^*fl/fl*^*;Mx1*-*Cre* and *Pten*^*fl/fl*^*;Mx1*-*Cre;ETV7Tg*^+*/WT*^ mice

Animals were housed in the St Jude Animal Resources Center with access to sterilized food and water ad libitum, and all experiments were approved by the Institutional Animal Care and Use Committee of St. Jude Children’s Research Hospital. B6.129S4fl*tm1Hwu*/J (*Pten*^*fl/fl*^) mice were kindly provided by Dr. Suzanne Baker. *ETV7Tg*^+*/WT*^ on the 129/CL57/B6 (129SvEv;C57Bl/6 mixed genetic background) were generated by backcrossing *ETV7Tg*^+*/WT*^ FVB mice onto wild-type 129/CL57/B6 mice for more than 10 generations prior to crossing them with *Pten*^*fl/fl*^*;Mx1*-*Cre*. 4–6-Week-old *Pten*^*fl/fl*^*;Mx1*-*Cre* and *Pten*^*fl/fl*^*;Mx1*-*Cre;ETV7Tg*^+*/WT*^ mice were injected intraperitoneally with seven doses of polyinosine–polycytidine (pIpC) (25 μg/g) every other day for 14 days to induce Cre expression as described previously (Yilmaz et al. 2006). Five days after pIpC injection, total BM was harvested and analyzed by colony-forming cell assays. The pIpC treated mice were observed daily and moribund mice were euthanized by CO_2_ inhalation. For pathologic diagnosis, the spleen, thymus, and sternum of sick mice were fixed in 10% neutral-buffered formalin and embedded in paraffin. All sections were stained with hematoxylin and eosin (H&E), and immunostained with anti-CD3 (Santa Cruz), anti-CD45R/B220 (BD Biosciences), and anti-myeloperoxidase (MPO; Dako) antibodies.

### Colony-forming cell (CFC) assay

BM cells were plated in methylcellulose-based media supplemented with 10 μg/mL insulin, 200 ng/mL human transferrin, 50 ng/mL mSCF, 10 ng/mL mIL-3, 10 ng/mL hIL-6, and 3U/mL erythropoietin (MethoCult M3434, StemCell Technologies) at a density of 10,000 cells per dish. The colonies were counted and pooled 10–14 days later, and re-plated into a secondary methylcellulose culture (MC2). This procedure was repeated for 4 rounds (MC4).

## Results

### ETV7 expression in normal human tissues

**Fig. 1:**
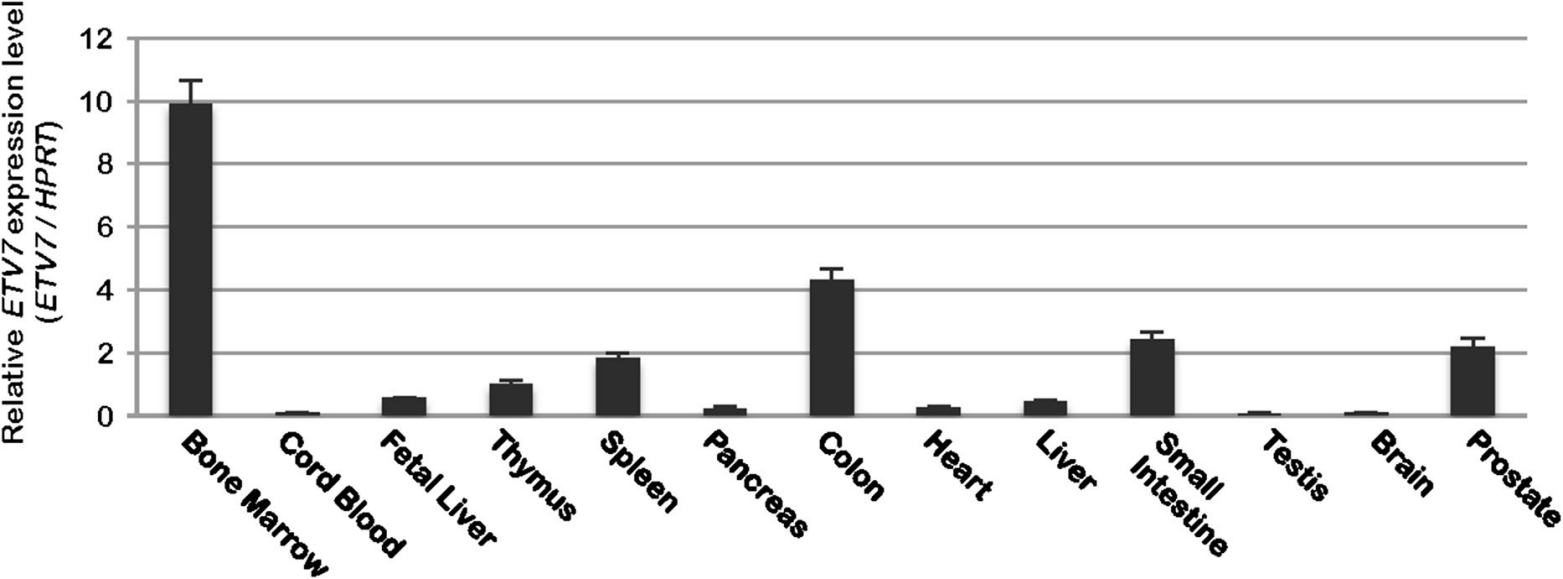
Expression level of *ETV7* mRNA in normal human tissues. Quantitative PCR was performed with cDNA libraries of various normal human tissues. The relative expression level of *ETV7* was normalized to *HPRT*

Previously we have shown by northern blotting that ETV7 is expressed in human bone marrow (BM) and fetal liver (Potter et al. 2000). To screen additional tissues for expression of ETV7, we performed quantitative PCR (q-PCR) using commercially available cDNA libraries from a variety of human tissues (Fig. 1). Although ETV7 is always expressed at low levels, we found the highest amount of ETV7 cDNA in BM and somewhat lower amounts in the colon, small intestine, prostate and spleen, while low levels of ETV7 cDNA were detected in pancreas, heart, brain, and testis libraries. This is largely in agreement with ETV7 RNA-seq data of different human tissues posted in the GTEx portal (https://www.gtexportal.org/home/gene/ETV7).

### Generation of ETV7 BAC transgenic mice

**Fig. 2:**
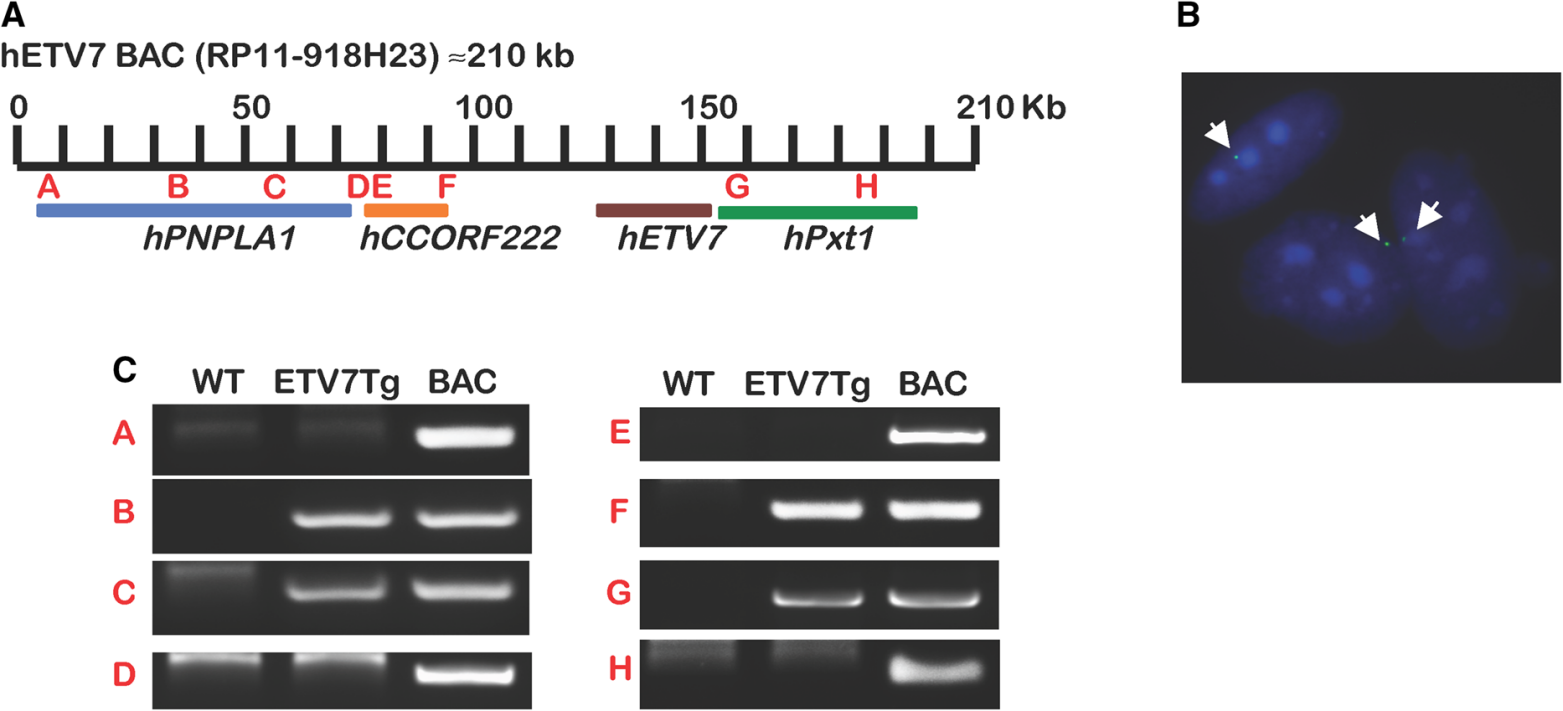
Design of the ETV7 BAC transgenic mouse. **a** Schematic representation of the ORFs present in human RP11-918H23 BAC DNA. **b** FISH detection of integrated BAC DNA showing a single signal in a proportion of tail fibroblasts. **c** Human gene specific primer locations (A–H) that were used to genotype *ETV7Tg*^+*/WT*^ (*ETV7Tg*) mice. PCR primers located in the first and last exons of all ORFs present in RP11-918H23 were used to map which sequences of the ETV7 BAC DNA had integrated in the host genome. Besides the *ETV7* gene, no productive human ORFs were present in the genomic DNA of the *ETV7Tg* mouse

Given that ETV7 is expressed in multiple human tissues, we wished to determine the potential functional consequences of ETV7 expression in *M. musculus*, which does not have the *Etv7* gene. We generated a mouse containing the human *ETV7* gene locus as part of a bacterial artificial chromosome (BAC) transgene. The use of BAC DNA to generate a transgenic mouse (BAC transgenic mouse) has been extensively documented and proven to be a useful approach (Moreira et al. 2007; Ristevski 2005). Unlike standard-type transgenes, large genomic transgenes have a much higher chance to include most if not all gene regulatory elements required for cell type specific expression of the transgene. Fertilized mouse oocytes were injected with RP11-918H23 BAC DNA containing the human *ETV7* gene locus (Fig. 2a). In total 55 F1 offspring were tested for presence of the transgene. PCR primers specific for sequences located 5 kb upstream and downstream of the *ETV7* gene identified three mice containing the whole gene. All three founders were mosaic for the transgene of which one generated *ETV7*-positive offspring. This mouse carried a single copy of the *ETV7* transgene as determined by quantitative PCR and FISH analysis (Fig. 2b). Next, we used additional PCR analysis to determine which portion of the BAC DNA was integrated. Primer pairs were used within the first and last exon of each open reading frame (ORF) contained within the BAC (Fig. 2c). A 70 kb portion containing the complete *ETV7* locus was detected (Fig. 2a, c; gels F and G). A second portion of BAC DNA was also detected, containing a roughly 30 kb internal fragment of the human patatin-like phospholipase domain containing 1 (*PNPLA1*) gene (Fig. 2a, c; gels B and C), but this region does not contain an ORF due to lack of exon 1 (Fig. 2c; position A). This indicated that *ETV7* was the sole intact ORF within the integrated human BAC DNA sequences.

### *ETV7Tg* mice have a normal phenotype and lifespan

**Fig. 3:**
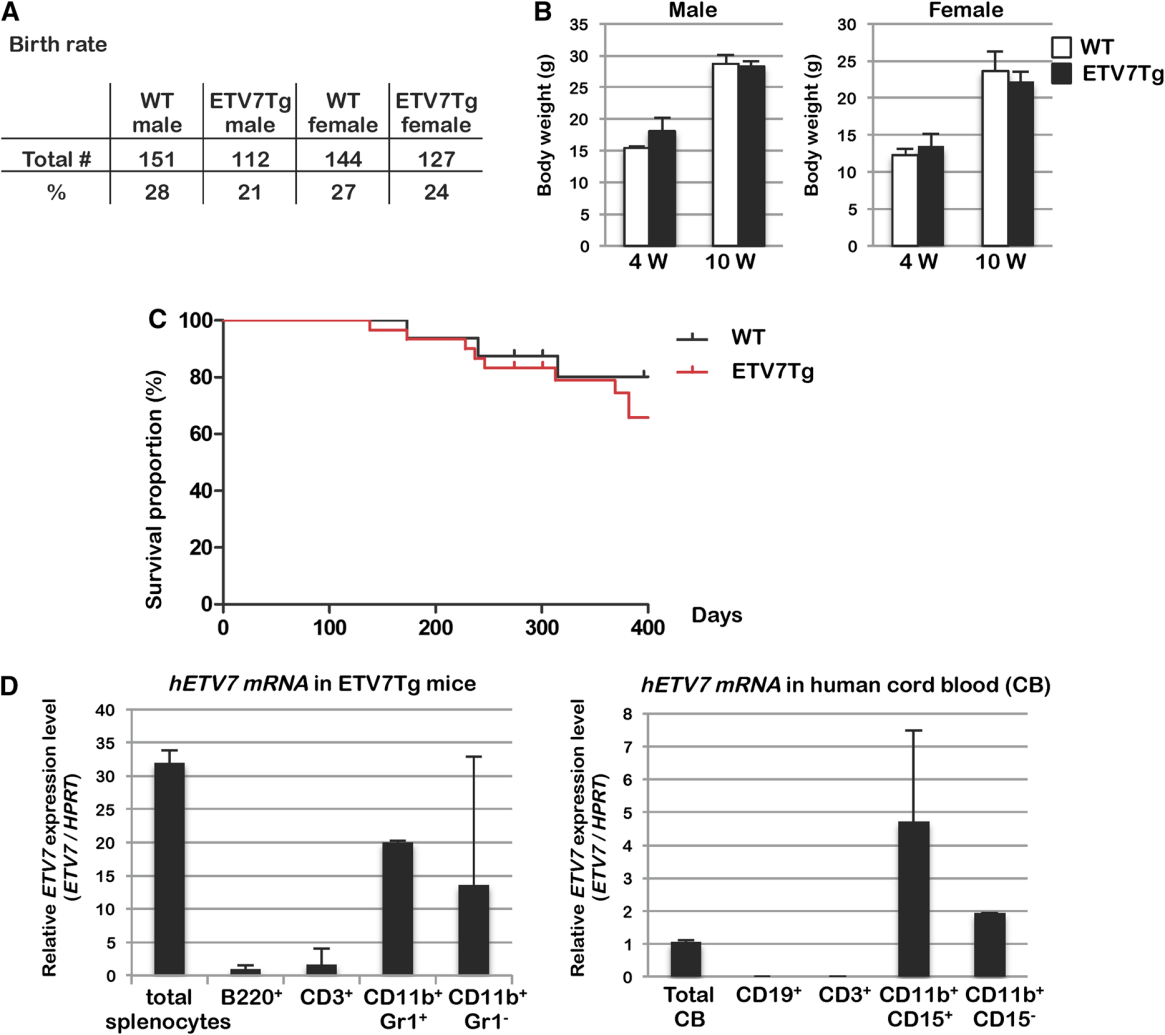
ETV7 BAC transgenic mice are fertile, develop normally, and remain tumor-free. **a** The table summarizes the birth rate of genotyped pups (n = 564) from breeding couples of wild-type (WT) females and *ETV7Tg*^+*/WT*^ (*ETV7Tg*) males. **b** The bar graph summarizes the body weight of 4 or 10-week-old WT or *ETV7Tg* mice. **c** Kaplan–Meier survival curves of WT (n = 16) and *ETV7Tg* (n = 30) mice on the FVB background over a period of 400 days. The mice with spontaneous rectal prolapse were euthanized for animal welfare reasons. None of these harbored tumors. **d** The relative expression level of *Etv7* in hematopoietic cells of *ETV7Tg* mice and humans was determined by qRT-PCR. Murine or human hematopoietic cell subtypes consisting of B-cells (B220^+^ or CD19^+^), T-cells (CD3^+^), and myeloid cells (Mac1^+^Gr1^−^ and Mac1^+^Gr1^+^ or Mac1^+^CD15^−^ and Mac1^+^CD15^+^) were sorted from *ETV7Tg* spleen or human cord blood, respectively

In this study, we used only hemizygous *ETV7Tg*^+*/WT*^ (*ETV7Tg*) transgenic mice. *ETV7Tg* mice were viable, produced normal sized litters, and had no discernable phenotype. FVB *ETV7Tg* mice were maintained by breeding *ETV7Tg*^+*/WT*^ with wild-type (WT) FVB mice, which produced a Mendelian distribution of the *ETV7* transgene in the offspring. This genetically confirmed the presence of a single integration site of the *ETV7* transgene. There was no male to female bias in the offspring in any of the litters (Fig. 3a). Also, there was no difference in body weight of transgenic mice compared with gender matched littermate controls both at 4 and 10 weeks of age (Fig. 3b). Animals were followed for 400 days to enable detection of long-term effects of ETV7 expression (Fig. 3c), but there was no difference in survival rate between transgenic and normal FVB mice. Therefore, we concluded that the *ETV7Tg* mice are not tumor-prone, despite the observation that a high percentage of human tumors overexpress ETV7, including 70% of childhood acute lymphoid and myeloid leukemia (Ross et al. 2003, 2004).

To examine the expression pattern of ETV7 in the hematopoietic system, we compared the expression level of ETV7 in hematopoietic tissues of *ETV7Tg* mice and humans. Murine or human B-cells, T-cells, and myeloid cells were isolated by fluorescence-activated cell sorting (FACS) from *ETV7Tg* splenocytes or human cord blood, respectively, and RNA of these cells was subjected to qRT-PCR analysis. As in humans, ETV7 expression was low in both B- and T-cells compared with that in myeloid cells of *ETV7Tg* mice (Fig. 3d). This result indicated that the ETV7 expression pattern in hematopoietic cells of *ETV7Tg* mimics that in humans.

### *ETV7Tg* mice express ETV7 in hematopoietic tissues

**Fig. 4:**
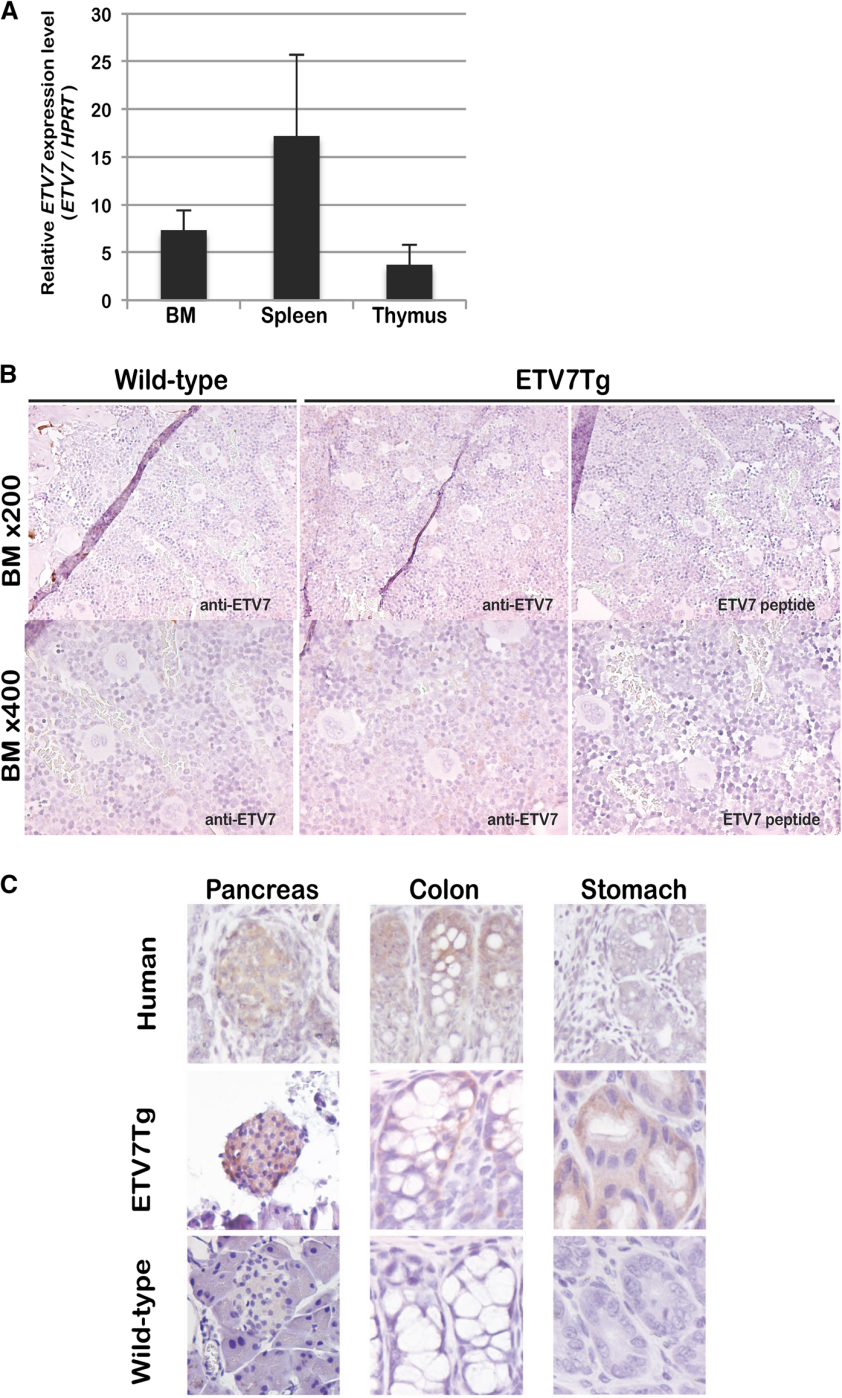
ETV7 BAC transgenic mice express ETV7 in a cell-type specific manner. **a** The relative expression level of *ETV7* in *ETV7Tg* hematopoietic tissues [bone marrow (BM), spleen, and thymus] was determined by qRT-PCR. **b** 8–12-Week-old healthy BM sections of WT and *ETV7Tg* mice were stained with anti-ETV7 antibody in the absence or presence of ETV7 blocking peptide, and counterstained with hematoxylin. **c** Anti-ETV7 staining of pancreas (left), colon (middle), and stomach (right) from human, *ETV7Tg* mice, and WT mice. ETV7 was detected in human and *ETV7Tg* islets of Langerhans, crypts of the colon and stomach, but not in these tissues of a WT mouse

To characterize the potential effect of ETV7 expression on the development of hematopoietic tissues, we determined the expression level of ETV7 in BM, spleen, and thymus of *ETV7Tg* mice (Fig. 4). 8–12-Week-old *ETV7Tg* mice and age matched littermate controls were sacrificed and tissue was harvested for qRT-PCR analysis and staining with anti-ETV7 antibody (Cardone et al. 2005) (Fig. 4). The expression of ETV7 was detected in all tissues with spleen showing the highest expression (Fig. 4a). Consistent with absence of the *Etv7* gene in mice, ETV7-positive cells were not detected in control BM cells (Fig. 4b, left). In contrast and compared with control BM, most of the *ETV7Tg* BM cells stained with the ETV7 antibody (Fig. 4b, middle) although mostly at low levels, indicating that the ETV7 protein is expressed at variable amounts. The ETV7 signal in *ETV7Tg* BM was absent after pre-incubation of the antibody with the ETV7-peptide against which the antibody had been raised (Fig. 4b, right), showing that our immunohistochemical staining is specific for ETV7 protein. In addition to hematopoietic tissues, pancreas, colon and stomach of age-matched littermate WT and *ETV7Tg* mice were stained with the ETV7 antibody. Human pancreas, colon and stomach were stained simultaneously. ETV7 was detected in pancreatic islets of Langerhans but not in exocrine tissues of *ETV7Tg* mouse or human pancreas (Fig. 4c, left), indicating that ETV7 expression is strictly cell-type specific. In agreement with this, the expression level of ETV7 mRNA in whole human pancreas was relatively low. Transitional cells in the colon displayed ETV7-positive staining as well as the cells within crypts of the stomach in both *ETV7Tg* mice and human (Fig. 4c, middle and right). The ETV7 staining of these tissues in WT littermates was always absent. Taken together, these data showed that the integrated *ETV7* gene locus in *ETV7Tg* mice is expressed at both the mRNA and protein level and is regulated in a tissue-specific fashion, reflecting the expression observed in normal human tissues.

### ETV7 expression does not discernably alter hematopoietic tissues in *ETV7Tg* mice

**Fig. 5:**
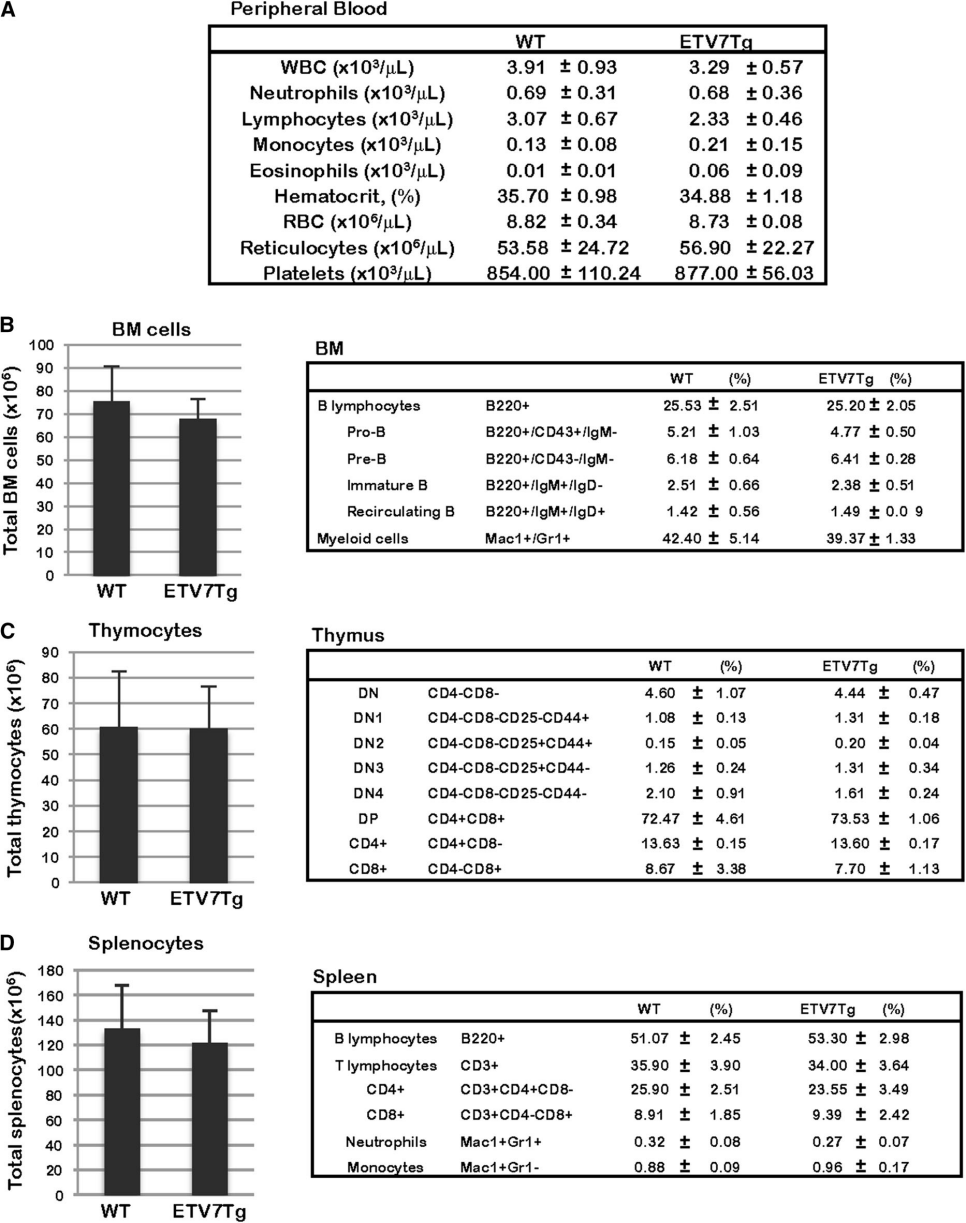
ETV7 does not affect the hematopoietic compartment of ETV7 BAC transgenic mice. **a** The panel shows the complete blood counts (CBC) of 9-week-old WT (n = 5) and *ETV7Tg* (n = 4) mice. **b** The bar graph on the left shows the total number of bone marrow cells in WT and ETV7 Tg mice, while the panel on the right shows the number of B-cells and myeloid cells in BM of 12-week-old WT (n = 3) and *ETV7Tg* (n = 3) male mice. Each cell subset was identified by its specific cell surface markers using flow cytometry. **c** The bar graph on the left shows the total number of T-cells in the thymus of 12-week-old WT (n = 3) and *ETV7Tg* (n = 3) male mice, while the panel on the right shows the numbers of different phenotypes as determined by flow cytometry. **d** The bar graph on the left shows the total number of splenocytes in 12-week-old WT (n = 3) and *ETV7Tg* (n = 3) male mice, while the panel on the right shows the number of B-cells, T-cells, and myeloid cells in the spleen of these mice

Despite expression of the *ETV7* transgene, no abnormalities were observed in any of the hematopoietic tissues by routine histological analysis (data not shown). To investigate hematopoietic homeostasis in more detail, we quantitatively examined the distribution of hematopoietic cell types in peripheral blood, BM, thymus, and spleen of *ETV7Tg* mice (Fig. 5). The relative proportion of leukocytes, erythrocytes, and thrombocytes in the peripheral blood of 9-week-old WT (n = 5) and *ETV7Tg* (n = 4) littermates was determined by complete blood count (CBC) (Fig. 5a). WT and *ETV7Tg* mice showed mostly similar CBC values although the average number of white blood cells (WBC) and lymphocytes was slightly lower in *ETV7Tg* mice. To further characterize the variety of lymphocytes and myeloid cells undergoing differentiation in vivo, BM cells, thymocytes, and splenocytes were freshly isolated from 12-week-old WT (n = 3) and *ETV7Tg* (n = 3) male mice, incubated with cell-surface specific antibodies, and analyzed by flow cytometry (FCM) (Fig. 5b–d). By counting the total number of mononuclear cells in hematopoietic tissues, we found the cellularity of BM, thymus, and spleen of *ETV7Tg* mice to be equivalent to that in control mice. In BM, a differential subset of B lymphocytes (B220^+^IgM^−^CD43^+^: progenitor (pro-) B, B220^+^IgM^−^CD43^−^: precursor (pre-) B, B220^+^IgM^+^IgD^−^: immature B, and B220^+^IgM^+^IgD^+^: Recirculating B) could be distinguished, while there was no difference in the relative proportion of each B cell type (Fig. 5b). In thymus, all subsets of T lymphocytes [CD4^−^CD8^−^: Double-Negative (DN), CD4^−^CD8^−^CD25^−^CD44^+^: DN1, CD4^−^CD8^−^CD25^+^CD44^+^: DN2, CD4^−^CD8^−^CD25^+^CD44^−^: DN3, CD4^−^CD8^−^CD25^−^CD44^−^: DN4, CD4^+^CD8^+^: Double-Positive (DP), CD4^+^CD8^−^: CD4^+^, and CD4^−^CD8^+^: CD8^+^] were similarly distributed in *ETV7Tg* and control mice (Fig. 5c). Also, *ETV7Tg* splenocytes showed a similar frequency of B220^+^ B-cells, CD3^+^ T-cells, and Mac1^+^Gr1^+^ or Mac1^+^Gr1^−^ myeloid cells as splenocytes of WT mice (Fig. 5d). Collectively, these results indicated that steady-state adult hematopoiesis in *ETV7Tg* mice was not altered by expression of the *ETV7* transgene.

### ETV7 enhanced cell proliferation of myeloid cells in vitro

**Fig. 6:**
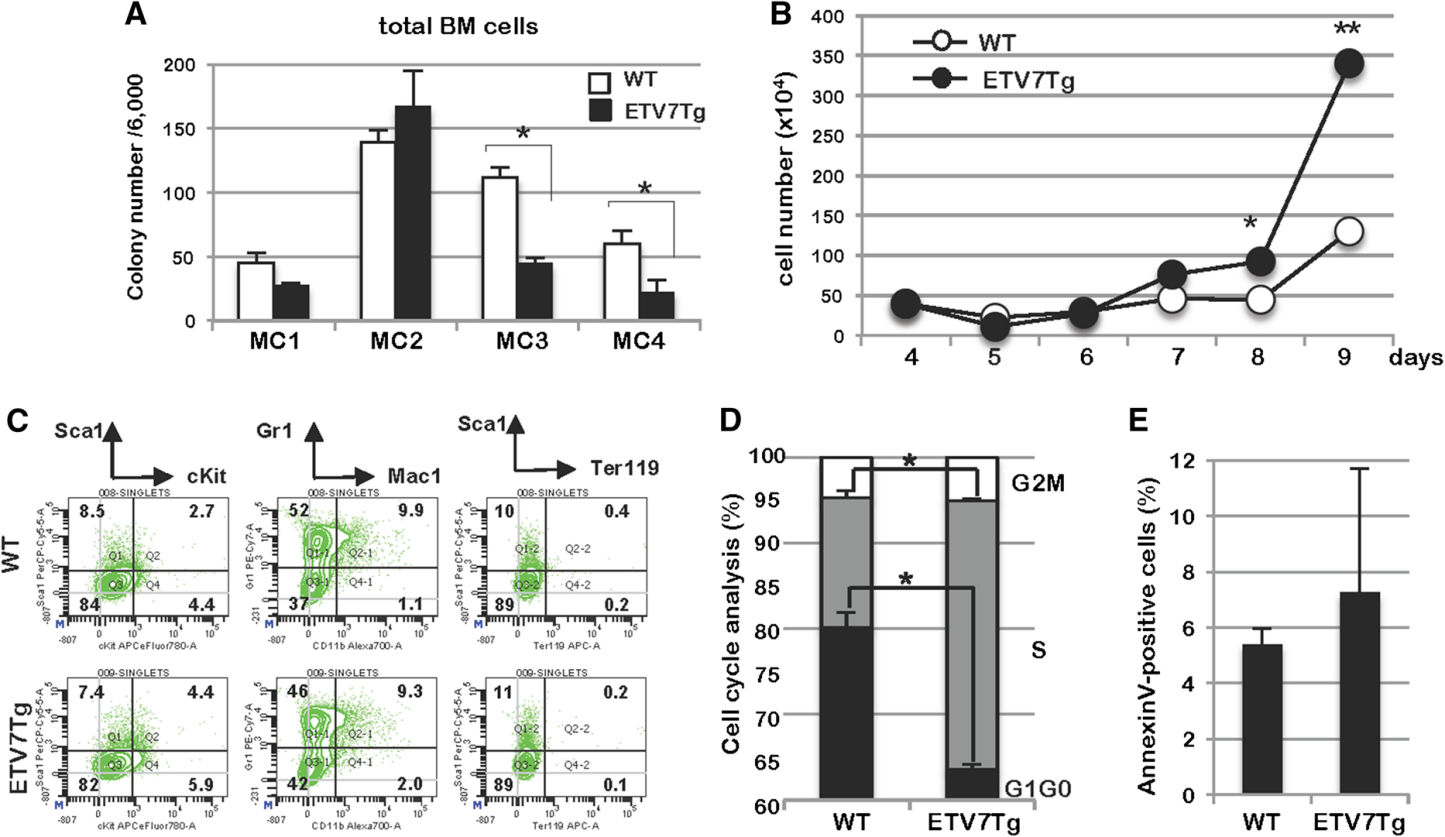
ETV7 enhances cell proliferation of BM-derived myeloid cells after long-term culture. **a** MethoCult assays were performed using total BM cells of 8-week-old WT and *ETV7Tg* mice. Colonies in the first plating (MC1) were counted at 10–12 days post seeding, followed by pooling and replating in new MethoCult medium for an additional 3 rounds (MC2–MC4). *p < 0.05. **b** Cell growth was examined of WT and *ETV7Tg* lineage-depleted (Lin^−^) BM cells in liquid culture in the presence of myeloid cytokines from Day 4–9 post seeding. *p < 0.05, **p < 0.01. **c** Lin^−^ BM cells cultured for 6 days were stained with the indicated antibodies and subjected to FCM analysis. **d** Cell cycle status was analyzed by flow cytometry at Day 19 of in vitro culture and the bar graph shows the proportion of cells in G_1_/G_0_, S, and G_2_/M phase of the cell cycle *p < 0.05. **e** The percentage of cells undergoing apoptosis at Day 19 of culture was determined by FCM analysis following AnnexinV/DAPI staining

Given that ETV7 can function as a hematopoietic oncogene, we next determined whether ETV7 changed the proliferation characteristics of myeloid cells in vitro. Total BM cells were freshly isolated from WT (n = 3) and *ETV7Tg* (n = 3) mice, pooled, and plated in methylcellulose medium at a density of 1 × 10^4^ cells/dish. The number of colonies consisting of CFU-E, CFU-GEMM, and CFU-GM were counted at Day 10–12 after seeding. The primary colony-forming units (CFUs) were counted (MC1), harvested and replated into new methylcellulose medium at the same density (MC2), and again scored after 10–12 days. This procedure was repeated four consecutive rounds (MC4). In the MC1 and MC2, we observed no difference in size, type, and number of WT and *ETV7Tg* CFUs (Fig. 6a). However, compared with WT control, *ETV7Tg* BM showed significantly decreased colony numbers in the MC3 and MC4, suggesting that there was reduced self-renewal activity of hematopoietic stem cells (HSCs) during the in vitro colony-forming assay. To test the proliferation of BM cells in vitro, lineage-depleted (Lin^−^) BM cells were cultured in liquid media supplemented with cytokines promoting myeloid cell growth (mSCF, mIL3, and hIL6). The culture was maintained for 30 days, by replacing the medium every 3 days. After 4 days of culture, *ETV7Tg* Lin^−^ BM cells grew significantly faster than control BM (Fig. 6b). At Day 6 of culture the cell type was determined by FCM analysis showing that almost half were Mac1^−^Gr1^+^ myeloid cells (Fig. 6c). Notably, the composition of the WT and *ETV7Tg* BM cultures was the same, suggesting that ETV7 did not alter myeloid-lineage differentiation in vitro. Because *ETV7Tg* myeloid cells exhibited a growth advantage, we compared their cell cycle status by flow analysis and their apoptotic index by Annexin V staining with that of WT myeloid cells after 19 days of liquid culture. In agreement with the enhanced cell proliferation, the percentage of cells in G_1_/G_0_ phase in *ETV7Tg* (63.62 ± 0.55%) was lower than that in control cultures (80.17 ± 1.75%), while the fraction of S phase *ETV7Tg* cells (31.34 ± 0.17%) was much higher (15.14 ± 0.74%) (Fig. 6d). On the other hand, there was no difference in the number of apoptotic cells between the two cultures (Fig. 6e). These results indicate that ETV7 accelerates cell cycle traverse of myeloid cells in vitro.

### ETV7 accelerates development of PTEN^Δ/Δ^ T-cell lymphoblastic leukemia in mice

**Fig. 7:**
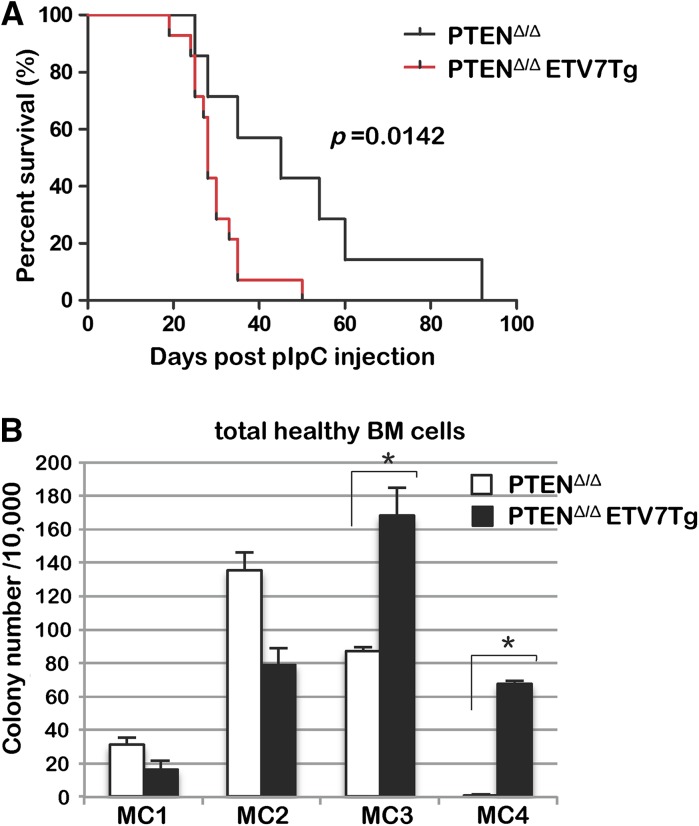
ETV7 accelerates the onset of PTEN^Δ/Δ^ T-cell lympho-leukemia. **a** Kaplan–Meier survival curves of *PTEN*^*Δ/Δ*^ (n = 10) and *PTEN*^*Δ/Δ*^*ETV7Tg* (n = 16) mice. The deletion of floxed-*PTEN* gene loci was induced by activation of Mx1-Cre recombinase by pIpC I.P. injection. **b** MethoCult assays were performed with myeloid cytokines using total BM cells of *PTEN*^*Δ/Δ*^ (n = 3) and *PTEN*^*Δ/Δ*^*ETV7Tg* (n = 3) mice, 2 days after pIpC injection. The colonies were counted and replated every 10–12 days

PTEN, a negative regulator of the oncogenic phosphoinositol-3-OH kinase (PI3K)-Akt pathway, is involved in cell proliferation, differentiation, survival, mobilization, and hematopoietic stem/progenitor cell function (Cully et al. 2006). Deletion of or inactivating mutations in the *PTEN* tumor suppressor gene have been found in childhood T-cell acute lymphoblastic leukemia (T-ALL) (Gutierrez et al. 2009). Within a few weeks conditional deletion of *Pten* in mice causes a myeloproliferative disease (MPD), which 4–6 months later develops into ALL or acute myeloid leukemia (AML) (Yilmaz et al. 2006; Zhang et al. 2006). To test the contribution of ETV7 to leukemogenesis in a *Pten*^*fl/fl*^ acute myeloid/lymphoid mouse model, we crossed *ETV7Tg*^+*/WT*^ mice with polyinosine–polycytidine (pIpC)-inducible conditional *Pten*^*fl/fl*^*;Mx1*-*Cre* mice. Within a few months after pIpC injection most *Pten*-deleted (PTEN^Δ/Δ^) and ETV7-positive/*Pten*-deleted (*PTEN*^*Δ/Δ*^*ETV7Tg*) mice succumbed to T-ALL and some to AML (Fig. 7a). ALL or AML was characterized as described previously (Yilmaz et al. 2006). Based on the pathological and histological analysis, there was no difference in tumor type and frequency of AML and ALL between the two genotypes. However, ETV7 greatly reduced the tumor-free lifespan of mice, indicating that ETV7 cooperatively accelerated the onset of Pten^Δ/Δ^ leukemogenesis. To investigate the oncogenic role of ETV7 on a hyperactivated PI3K/Akt background, colony-forming assays were performed using normal total BM cells of PTEN^Δ/Δ^ and PTEN^Δ/Δ^*ETV7Tg* mice 5 days post pIpC treatment (Fig. 7b). Contrary to the presence of ETV7 alone in Fig. 5a, PTEN^Δ/Δ^*ETV7Tg* BM cells produced a greater number of colonies in MC3 and MC4 as did PTEN^Δ/Δ^ BM cells, suggesting that ETV7 enhanced the colony-forming activity of Pten^Δ/Δ^ hematopoietic stem/progenitor cells. These results indicated that moderate expression of ETV7 collaborates with the PTEN/PI3K/Akt pathway to develop leukemia in Pten^Δ/Δ^ mice.

## Discussion

ETV7 has been implicated in both human and mouse hematopoietic malignancy. Elevated ETV7 expression was observed in 70% of pediatric ALL/AML patients and overexpression of ETV7 inhibited monocytic differentiation in human cell lines (Kawagoe et al. 2004). In the mouse, retroviral transduction of ETV7 in BM caused myeloproliferative disease and in combination with *Myc* over expression accelerated B-cell lymphoma development (Cardone et al. 2005; Carella et al. 2006). However, these studies were not intended to identify the role of endogenous ETV7 in normal development and tumorigenesis because the oncogenic capabilities of ETV7 became apparent upon forced overexpression of ETV7 in murine BM and transformed human cell lines. Indeed, qRT-PCR showed that the expression level of ETV7 driven by the *MSCV* promoter in BM was over 1,000,000-fold higher than that in *ETV7Tg* BM (data not shown). It is curious that in spite of its high level of conservation among vertebrates the *Etv7* gene locus was lost in part of the rodents including the mouse. This has limited the study of both its physiological and tumorigenic function in a mammalian model in vivo. In this study, we first established a unique transgenic mouse model, which carries a single copy of the human *ETV7* gene and its regulatory elements (10 kb upstream sequences, 33.7 kb representing ETV7 and 26 kb of downstream sequences). Unfortunately, we managed to only obtain a single transgenic line as the other two chimeric transgenic founders never gave germline transmission of the transgene. Given that all three founders were chimeric, we suspect that after oocyte injection the BAC might be toxic during the transient phase of gene expression before integration into the genome, resulting in only chimeric offspring. Therefore, a limitation of this study is that our data and conclusions are derived from a single transgenic mouse line.

ETV7 expression in hematopoietic tissues in *ETV7Tg* mice was similar to that in humans, and the hematopoietic system developed normally without any skewing of cell types, or symptoms of disease. However, once our *ETV7Tg* mice were crossed with oncogenic *Pten*^*fl/fl*^*;Mx1*-*Cre* mice, ETV7 accelerated PTEN^Δ/Δ^ leukemia. This result demonstrates the potential of the *ETV7Tg* mouse to serve as a more appropriate surrogate to study ETV7-positive human malignancies.

Recently we reported a potential in vivo function of ETV7 using zebrafish (Quintana et al. 2014). ETV7 knock-down in these animals disrupted red blood cell development indirectly through inhibition of the cholesterol synthesis pathway. Based on these results we speculated that appropriately regulated but nonetheless ectopically expressed human ETV7 in the mouse might affect red blood cell development or perturb other developmental aspects of hematopoiesis. However, we could not find any alteration in adult hematopoiesis or an altered distribution of hematopoietic cells in *ETV7Tg* mice. This could be the result of an as yet unidentified compensatory mechanism, or alternatively, low level of strictly regulated ETV7 expression might be insufficient to induce a discernable phenotypic change. Human hematopoietic cells are able to express ETV7 without detrimental effects, in contrast to the increased expression found in a large proportion of their malignant counterpart, further underwriting the potential impact of altered expression levels of ETV7. To establish the biological significance of the *ETV7Tg* mice for the analysis of ETV7 in normal ontogenesis and tumorigenesis, it is crucial to isolate the ETV7-interacting factors and identify its direct or indirect transcriptional targets. Moreover, besides hematopoietic tissues, it will be interesting to investigate the physiological or tumorigenic role of ETV7 in other tissues such as colon and small intestine that showed relative high expression of ETV7 in both normal human tissues and the *ETV7Tg* mouse.

Although *ETV7Tg* mice showed no obvious phenotype in hematopoietic tissues in vivo, *ETV7Tg* myeloid cells grew faster than control cells in in vitro culture. This was the result of accelerated cell cycle traverse rather than inhibition of apoptosis (Fig. 6b, d, e). Since *ETV7Tg* mice need to acquire other genetic and/or epigenetic mutations for transformation, the enhanced cell proliferation is likely to be an essential step in the accumulation of genetic mutations, perhaps as a result of increased replicative stress. *ETV7Tg* primitive myeloid progenitors showed reduced colony-forming activity during serial methylcellulose assays (Fig. 6a, MC3 and MC4), suggesting that the colony-forming activity of progenitors and/or the self-renewal activity of hematopoietic stem cells (HSC) is impaired in *ETV7Tg* BM. The ETS transcription factor TEL/ETV6, a frequent target of chromosomal translocation in human leukemia (Bohlander 2005), is known to be a selective and essential regulator of HSC survival in mice (Hock et al. 2004). Given that ETV6 and ETV7 have opposite biological functions and can physically interact via their PNT domains (Fenrick et al. 2000; Kawagoe et al. 2004; Potter et al. 2000; Rompaey et al. 2000), it is conceivable that ETV7 interferes with ETV6’s function in HSC maintenance. However, during constitutively activated PI3K/Akt signaling due to loss of *Pten*, ETV7 enhanced myeloid colony-formation compared with Pten^Δ/Δ^ control cells, suggesting that ETV7 works as a positive regulator in absence of the *Pten* tumor suppressor.

In addition to *PTEN*^*fl/fl*^ mice, we also crossed *ETV7Tg* mice with *Arf*^−*/*−^(Arf^−/−^) and *Ink4aArf*^−*/*−^(*Ink4aArf*^−*/*−^) mice to generate *Arf*^−*/*−^*ETV7Tg*^+*/WT*^ (*Arf*^−*/*−^*ETV7Tg*) and *Ink4aArf*^−*/*−^*ETV7Tg*^+*/WT*^ (*Ink4aArf*^−*/*−^*ETV7Tg*) double mutant mice, respectively. Intriguingly, ETV7 slightly shortened the tumor onset of *Arf*^−*/*−^ mice but *Ink4aArf*^−*/*−^ and *Ink4aArf*^−*/*−^*ETV7Tg*^+*/WT*^ mice showed completely overlapping survival curves (Supplemental Fig. 1). All mice developed a variety of tumors as described previously (Kamijo et al. 1997; Serrano et al. 1996) and ETV7 did not affect the tumor spectrum of *Arf*^−*/*−^ and *Ink4aArf*^−*/*−^ mice. Nonetheless, 1 out of 26 *Arf*^−*/*−^*ETV7Tg* mice succumbed to myeloid leukemia and hemangiosarcoma, which has never been reported in *Arf*^−*/*−^ control mice. These results suggest that ETV7 expression is functionally comparable with reduced pRb cell cycle control, which agrees with the observation that ETV7 accelerates cell cycle traverse in myeloid cells.

In conclusion, we provide evidence that our ETV7 BAC transgenic mouse model developed normally and did not show any apparent phenotype but showed increased tumor incidence when crossed onto a tumor-prone mouse background. The *ETV7Tg* mouse is therefore a more faithful cancer animal model to investigate ETV7-associated human tumors. We have started crossing the *ETV7Tg* mice with various other cancer mouse models to determine if ETV7 also accelerates tumorigenesis in those models in vivo. We believe that our *ETV7Tg* mouse not only enables us to more faithfully model human ETV7-associated tumorigenesis in vivo but also provides a preclinical model with which to test efficacy of future drugs directed against ETV7-positive human cancers.

## Electronic supplementary material

Below is the link to the electronic supplementary material.

Supplementary Figure 1 Effect of ETV7 expression on the tumor incidence in *Arf*^*−/−*^ and *Ink4aArf*^*−/−*^ mice. *ETV7Tg* mice were crossed with *Arf*^*−/−*^ or *Ink4aArf*^*−/−*^ mice and single and double (*Arf*^*−/−*^*ETV7Tg* and *Ink4aArf*^*−/−*^*ETV7Tg*) transgenic offspring was maintained till all mice had succumbed to cancer. A) Kaplan–Meier survival curve of *Arf*^*−/−*^ single and *Arf*^*−/−*^*ETV7Tg* double transgenic mice. B) Kaplan–Meier survival curve of *Ink4aArf*^*−/−*^ single and *Ink4aArf*^*−/−*^*ETV7Tg*^*+/WT*^ double transgenic mice (TIFF 1587 kb)
