# Supplementary figures and images for: Establishment of a transgenic mouse to model ETV7 expressing human tumors
Source: Transgenic Res. 2018 Nov 27;28(1):115–28. doi: 10.1007/s11248-018-0104-z (PMC6353817; doi:10.1007/s11248-018-0104-z)

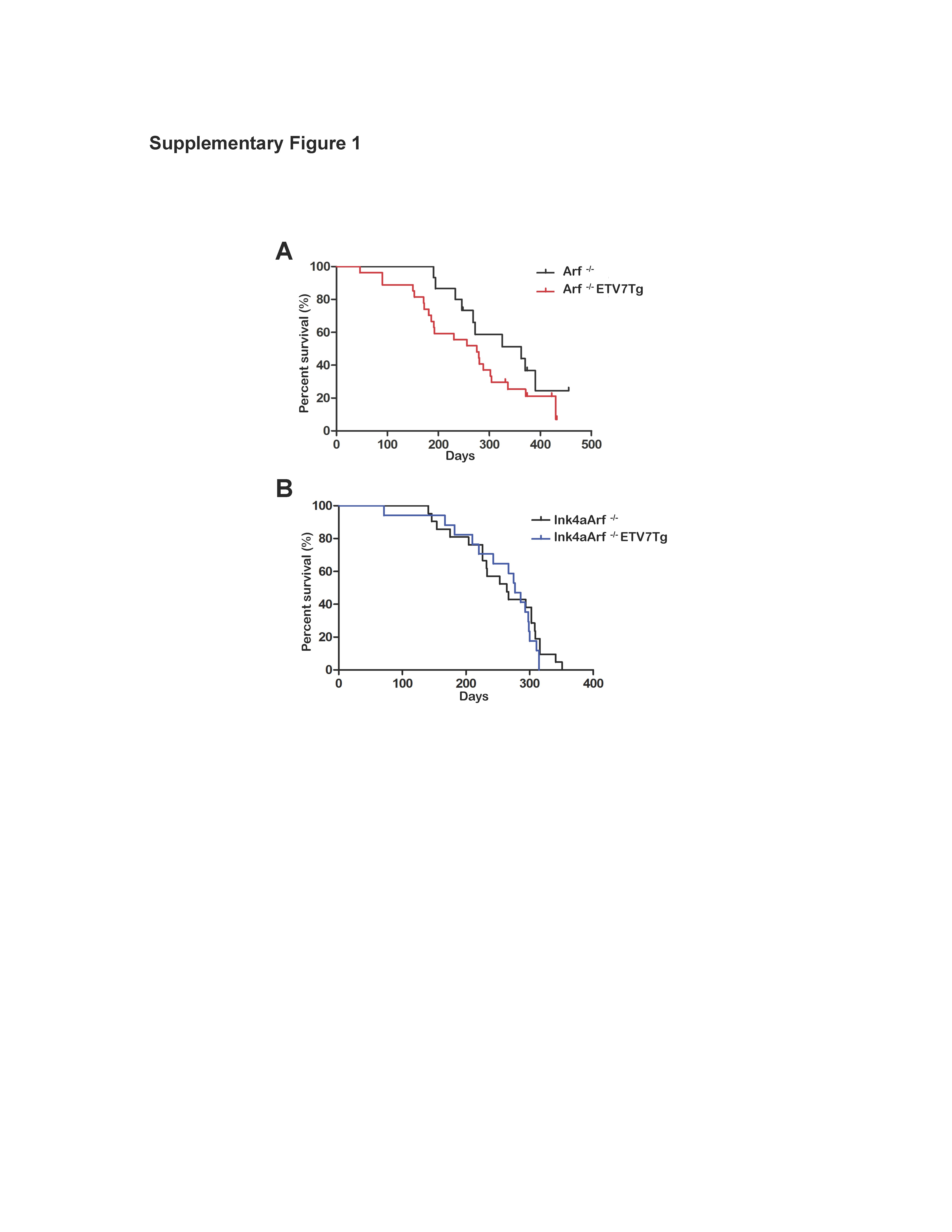

Supplement: Supplementary file 1 — Effect of ETV7 expression on the tumor incidence in Arf−/− and Ink4aArf−/− mice. ETV7Tg mice were crossed with Arf−/− or Ink4aArf−/− mice and single and double (Arf−/−ETV7Tg and Ink4aArf−/−ETV7Tg) transgenic offspring was maintained till all mice had succumbed to cancer. A) Kaplan–Meier survival curve of Arf−/− single and Arf−/−ETV7Tg double transgenic mice. B) Kaplan–Meier survival curve of Ink4aArf−/− single and Ink4aArf−/−ETV7Tg+/WT double transgenic mice (TIFF 1587 kb) [file 11248_2018_104_MOESM1_ESM.tiff]
